# Research on urban tree classification method based on YOLO-CNGD

**DOI:** 10.3389/fpls.2026.1754458

**Published:** 2026-02-25

**Authors:** Cunjin Zhang, Mei Liu, Xinglong Liu, Zhixin Gu

**Affiliations:** Computer and Control Engineering College, Northeast Forestry University, Harbin, China

**Keywords:** CBAM attention mechanism, remote sensing image, urban tree classification, YOLO-CNGD, YOLOv11n deep learning

## Abstract

Accurate classification of urban tree species is fundamental for urban green space management and ecological assessment. To address the challenges of small and overlapping tree crown detection in high-resolution remote sensing imagery, this study proposes YOLO-CNGD, a novel framework based on YOLOv11n. The key enhancements include the integration of the Convolutional Block Attention Module (CBAM) for refined feature representation, the adoption of the Normalized Wasserstein Distance (NWD) loss for robust small-object localization, the incorporation of Deformable Convolution v3 (DCNv3) to adapt to irregular shapes, and the replacement of standard convolutions with GhostConv for a lightweight design. Experiments on a self-built urban tree dataset show that YOLO-CNGD achieves a precision of 94.8%, a recall of 91.1%, and an mAP@0.5 of 93.7%. The model balances accuracy and efficiency, showing great potential for large-scale automated urban tree inventory.

## 1 Introduction

Integrating high-resolution remote sensing satellite imagery with deep learning algorithms enables precise analysis of urban tree species, quantity, distribution, boundaries, locations, and canopy extents. This approach enables real-time monitoring of urban tree growth and spatial distribution, supports species-specific distribution evaluation, and informs urban greening optimization and biodiversity conservation.

Over the past five years, the classification and detection of small-target trees in urban remote sensing imagery have seen remarkable progress. In 2020, Liu et al. (2020) improved a Convolutional Neural Network (CNN) model under the TensorFlow framework for the automatic identification of seven tree species. They employed the Adaptive Moment Estimation (Adam) optimizer with an exponentially decaying learning rate, incorporated L2 regularization into the cross-entropy loss function to penalize weights, and applied Dropout along with the Rectified Linear Unit (ReLU) activation function to prevent overfitting. In 2022, Liu et al. (2022) utilized four different types of point cloud deep learning models to classify and identify individual trees from eight species. All models, except PointNet, achieved classification accuracy exceeding 0.90.

In 2023, Vermeer et al. (2023) proposed a deep learning model for tree species classification using only LiDAR data. Based on a U-Net architecture for segmenting LiDAR images, the model was trained with a focal loss function to handle weakly labeled data, achieving an F1-score of 0.70. Also in 2023, Velasquez-Camacho et al. (2023) applied deep learning algorithms to detect, count, and locate urban trees, successfully identifying 79% of street trees. In 2024, Honkavaara et al. (Turkulainen et al., 2023) acquired RGB, multispectral, and hyperspectral imagery via drones. Their results demonstrated that the 2D-3D-CNN model performed best in classifying infested trees using hyperspectral data, with an F1-score reaching 0.742.

In the same year, Liu et al. (2024) proposed the YOLOv7-KCC model for tree species classification. By replacing standard convolutions with CoordConv and integrating the Convolutional Block Attention Module (CBAM) into the network, their model achieved a mean Average Precision (mAP) of 98.91%.

Recent studies in 2024 and 2025 have further highlighted the necessity of deep learning for fine-grained urban forestry. For instance, Satama-Bermeo et al. (2025) demonstrated that while YOLOv8 and the newly released YOLOv11 exhibit superior detection speeds, their performance on small, overlapping tree crowns in dense urban environments remains suboptimal without specific architectural modifications. Similarly, Zhao and Chen (2025) emphasized that accurate mapping in heterogeneous urban landscapes (e.g., Nanjing) requires overcoming the feature loss problem common in down-sampling processes. Furthermore, advanced lightweight models like SEMA-YOLO (Liu and Yang, 2025) and SRM-YOLO (Chen and Wang, 2025) have been proposed to tackle small object detection in remote sensing, validating the trend towards integrating attention mechanisms and multi-scale adaptations.

While existing models primarily focus on improving classification accuracy, they often underutilize the rich spatial and spectral information inherent in high-resolution remote sensing imagery (Ayrey et al., 2017). Despite these advancements, classifying urban trees in high-latitude cities like Harbin presents unique challenges. High-density building shadows often distort spectral signatures, and the extreme phenological similarity between *Salicaceae* species (e.g., Poplar and Willow) during the peak growth season leads to significant inter-class confusion. Current SOTA models often lack the specific mechanisms required to disambiguate these fuzzy boundaries under complex illumination. To bridge these gaps, we present YOLO-CNGD, a comprehensively modified YOLOv11n architecture. It is specifically designed to enhance feature representation for small urban tree crowns through synergistic integration of attention (CBAM), a tailored loss function (NWD), deformable convolutions (DCNv3), and lightweight operations (GhostConv).

## 2 Materials and methods

### 2.1 Study subjects and classification criteria

Harbin (45°42′–46°28′N, 126°29′–130°01′E) is located in the central-southern part of Heilongjiang Province and falls within the warm temperate continental monsoon climate zone. With an urban tree coverage rate of approximately 40%, the city experiences extremely low temperatures in winter, frequently accompanied by severe cold conditions (Jin et al., 2019). The vegetation primarily consists of deciduous and coniferous tree species, with dominant varieties including elm, willow, pine, poplar, and birch.

The theoretical basis for species classification integrates geometric characteristics and spectral analysis. *Ulmus* (elm) exhibits an irregularly rounded crown with relatively uniform foliage distribution. *Salix* (willow) is characterized by pendulous branches and an oblong or elliptical crown with a loosely defined margin. *Pinus* (pine) typically presents a conical or umbrella-shaped crown featuring dense foliage and a serrated silhouette, a key identifier of coniferous traits. *Populus* (poplar) displays a tall, columnar crown with a flattened apex. *Betula* (birch) is distinguished by its whitish bark and an ovate crown exhibiting a grayish-green hue.

Manual classification relying solely on geometric features is susceptible to subjective bias and inconsistency. Therefore, spectral feature analysis was performed using TIF-format remote sensing imagery, incorporating the blue, green, red, and near-infrared channels along with the Normalized Difference Vegetation Index (NDVI). NDVI is an indicator for assessing vegetation vigor and coverage, calculated from the reflectance in the near-infrared and red bands of remote sensing images (Rezatofighi et al., 2019). Its values range from -1 to 1, and an NDVI value greater than 0.6 typically indicates dense and healthy tree cover. The calculation formula is shown in **Equation 1**.

(1) $$NVDI=\frac{NIR-Red}{NIR+Red}$$

Here, NIR represents the reflectance in the near-infrared band, and Red denotes the reflectance in the red band.

The spectral reflectance characteristics and vegetation indices of the five tree species are summarized in **Table 1**. The wavelength ranges for the spectral bands are defined as follows: blue (450–500 nm), green (500–600 nm), red (600–700 nm), and near-infrared (700–900 nm).

**Table 1:** Spectral reflectance and vegetation indices.

Tree species	Blue light reflectance	Green light reflectance	Red light reflectance	NIR reflectance	NDVI
Elm	8%-12%	15%-20%	8%-12%	45%-55%	0.67
Willow	5%-10%	15%-25%	5%-10%	50%-60%	0.74
Pine	5%-10%	10%-15%	5%-10%	40%-50%	0.70
Poplar	5%-10%	15%-25%	5%-10%	50%-60%	0.74
Birch	10%-15%	15%-20%	10%-15%	40%-50%	0.58

### 2.2 Data preprocessing

The remote sensing imagery for the Harbin area, Heilongjiang Province, China, was acquired from Google Earth Engine and exported in GeoTIFF format to constitute the dataset. The images were captured on August 10, 2022, with a sensor altitude of 280 meters above sea level. Using the Python Pillow library, the TIF images were converted to PNG format. The final dataset comprises 2,500 images, each with a pixel size of 640 × 640.

The image data were annotated using Labelme. This tool saves the annotation information in JSON format, which is structured, easy to store, and machine-readable. The entire JSON file constitutes a dictionary-like structure, where various metadata elements are stored in key-value pairs. For instance, the “imageWidth” and “imageHeight” fields represent the width and height of the image in pixels, respectively.

To ensure the reliability of the dataset, we conducted a rigorous field verification campaign in August 2022. We randomly selected 500 samples from the annotated dataset, covering all five tree species, and verified their ground truth categories using a handheld GPS device (accuracy ±1m) and field photography. This on-site validation confirmed that our expert visual interpretation achieved an accuracy of over 96%, with minor corrections applied primarily to distinguish between young *Populus* and *Salix* trees in shaded areas.

To enhance model performance and generalization capability, ensuring its robustness in complex and varied image recognition tasks, data augmentation was performed through image rotation and mirror flipping. This process simulates the appearance of images from different perspectives. Mirror flipping includes both horizontal and vertical flipping, which increases data diversity and exposes the model to a wider range of directional variations in image samples.

**Figure 1:**
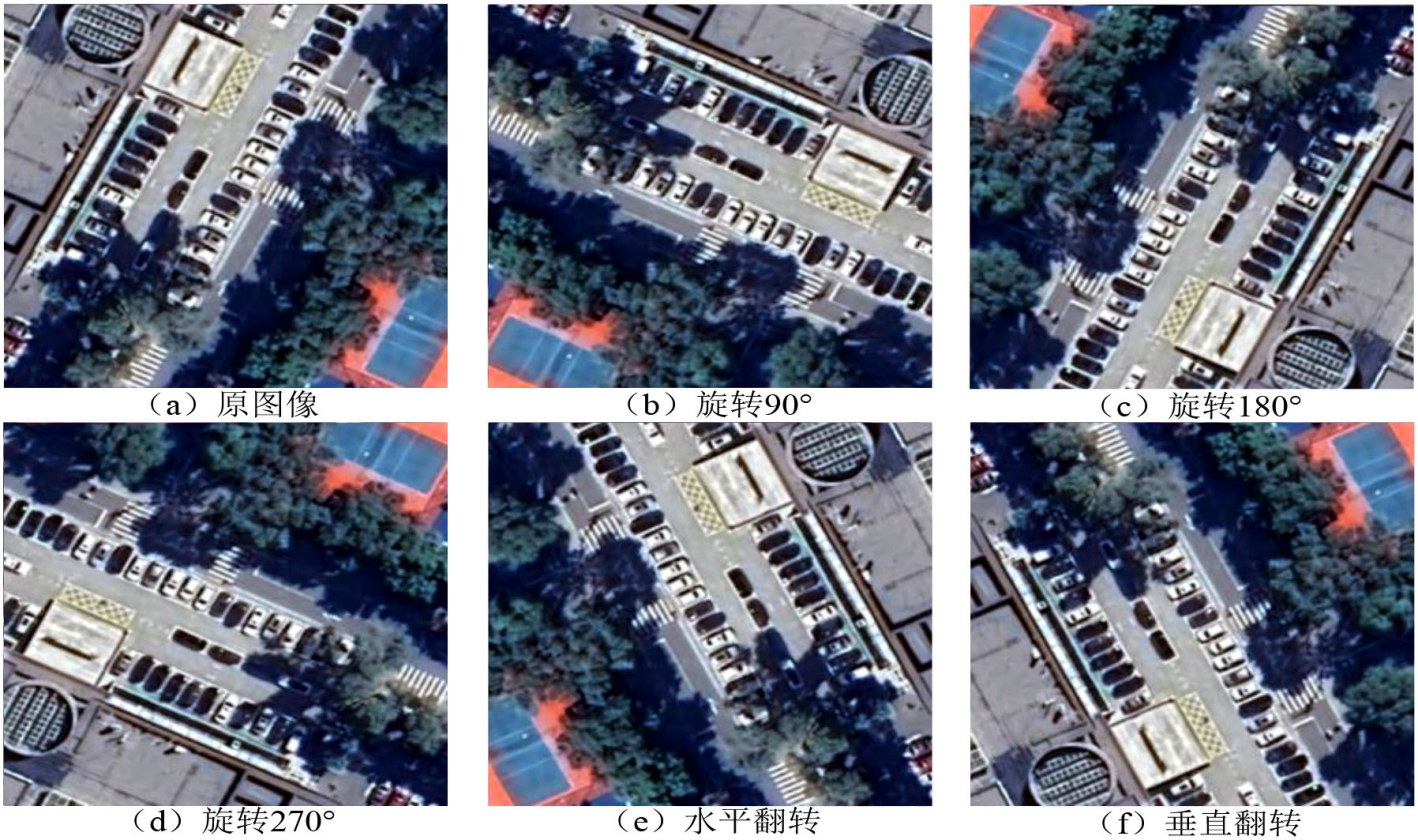
Example of data preprocessing.

Since both rotation and flipping significantly alter the position and orientation of target objects in the image, it is essential to update the corresponding annotation information promptly and accurately. The bounding boxes must be adjusted accordingly to ensure precise alignment with the transformed objects. An example of the data preprocessing procedure is illustrated in **Figure 1**. To ensure the integrity of model evaluation and prevent data leakage, the original dataset of 2,500 images was first split into training, validation, and test sets in a ratio of 8:1:1. Subsequently, data augmentation (rotation and flipping) was applied exclusively to the training set, expanding it to 15,000 images, while the validation and test sets remained unaugmented to represent real-world scenarios.

### 2.3 Model and methods

**Figure 2:**
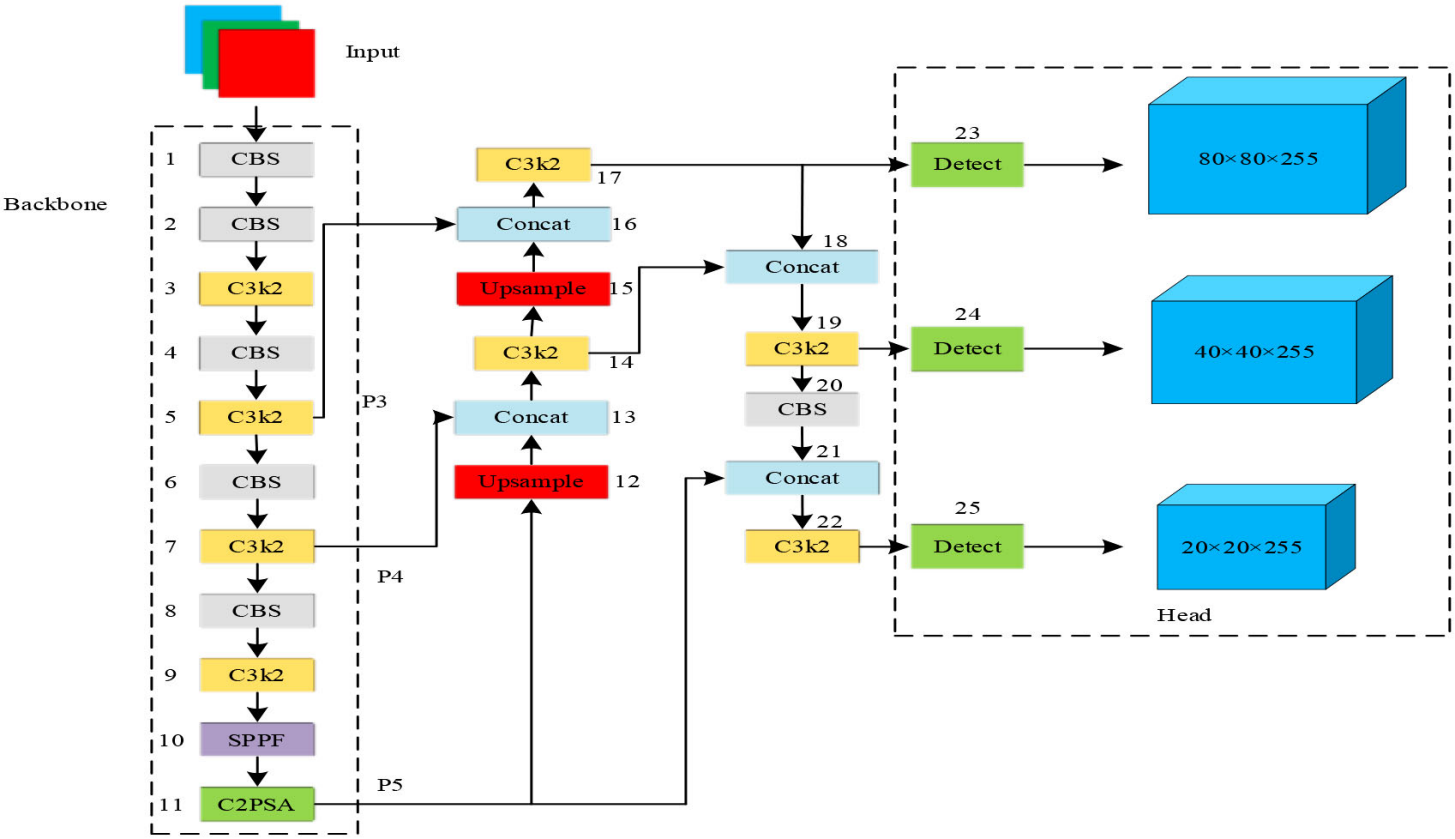
Architecture of the YOLOv11n model.

The YOLOv11n architecture (**Figure 2**) primarily consists of three core components: a Backbone, a Neck, and a Head.

The backbone network extracts hierarchical feature representations from the input image, and this process begins with a series of Convolution-Batch Normalization-SiLU (CBS) modules. Within these modules, the convolutional layer performs feature extraction, utilizing kernels of varying sizes and strides to capture local information at different scales. The Batch Normalization (BN) layer then normalizes the output of the convolution, accelerating model convergence and enhancing its generalization ability. Finally, the SiLU activation function introduces non-linear transformations, which augment the model’s expressive capacity and enable it to learn more complex patterns.

The Residual Connections facilitate the learning of residual information via skip connections, which mitigates the vanishing gradient problem in deep network training and enables the network to excavate more profound features. Furthermore, Group Convolution operates independently across multiple branches, significantly reducing both parameter count and computational overhead. This synergistic combination allows the subsequent C3k2 module to efficiently extract deep image features while minimizing computational cost and model parameters. Consequently, the design not only maintains detection accuracy but also enhances operational efficiency in resource-constrained environments.

The backbone network further incorporates a Spatial Pyramid Pooling-Fast (SPPF) module and a C2PSA module. The SPPF module is designed to enhance feature extraction capabilities. The input feature map first undergoes a preliminary transformation via a convolutional layer. It is then fed into three parallel max-pooling layers, which perform down-sampling at different scales to generate multi-scale features. These multi-scale features, along with the original input features, are concatenated, effectively integrating fine-grained details with broader contextual information. The fused features are subsequently refined through another convolutional layer to better suit the requirements of the object detection task.

**Figure 3:**
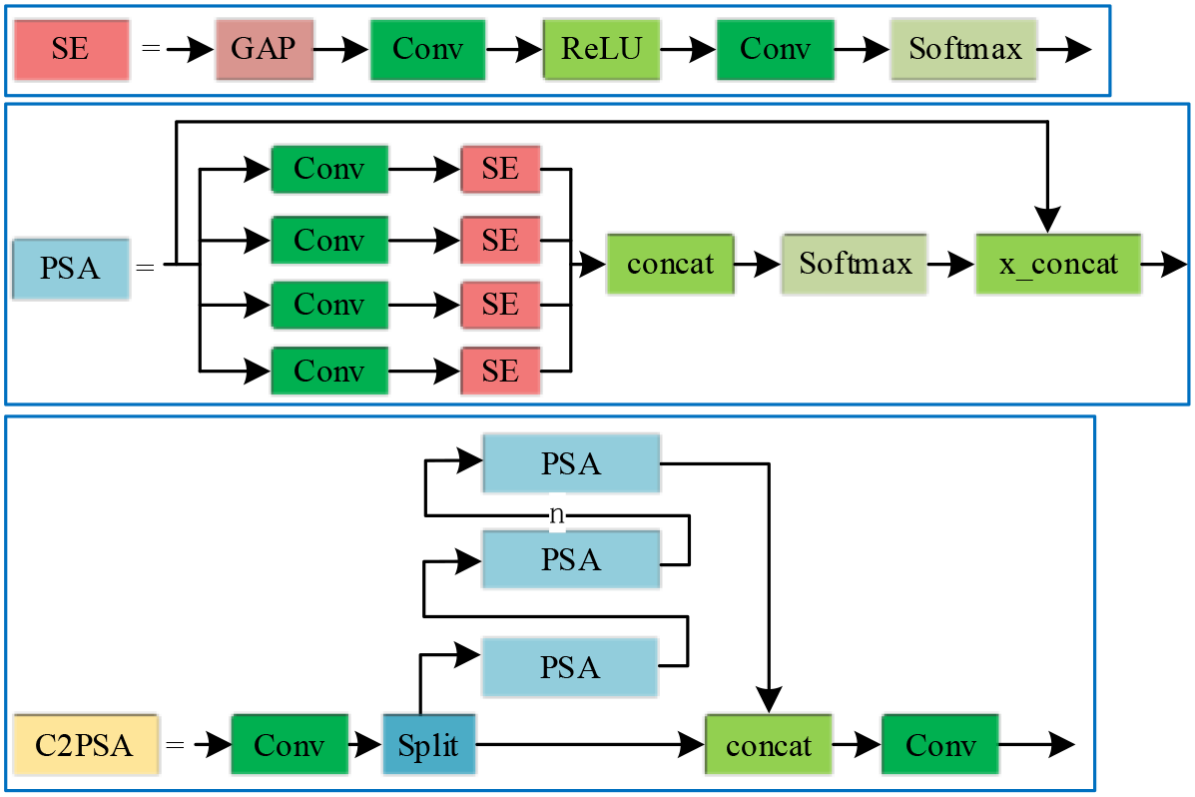
Architecture of the proposed C2PSA module.

The C2PSA module is constructed based on the Squeeze-and-Excitation (SE) module and the Pyramid Split Attention (PSA) module. The input feature map is first processed by a convolutional layer for initial transformation. A split operation then distributes the features into multiple branches, each of which is processed by a PSA sub-module. A structural diagram of the C2PSA module is presented in **Figure 3**.

The C2PSA module employs a multi-step, multi-dimensional feature processing strategy to more effectively capture subtle inter-class distinctions. Specifically, feature outputs from multiple parallel PSA submodules are first fused via a concatenation layer. The combined features then pass through a Softmax operation and are further aggregated through feature concatenation (x_concat), thereby enhancing the modeling of spatial dependencies. Subsequently, the fused features are refined and integrated by a convolutional layer. This structured approach enables the C2PSA module to sensitively discern fine-grained differences among target categories.

The primary function of the neck network is to integrate the hierarchical feature maps generated by the backbone, thereby constructing a feature pyramid rich in semantic information and multi-scale representations. Through iterative upsampling and concatenation operations, it progressively enhances the discriminative power and robustness of the features, ultimately providing superior input for the detection head.

The head network takes the fused feature maps from the neck as input. It then processes them through a series of dedicated Detect modules to produce the final detection outcomes. Each Detect module typically consists of convolutional layers for further feature refinement and transformation, followed by a prediction layer that estimates both the class probabilities and spatial coordinates of the objects.

The head network of YOLOv11n generates detection outputs across three distinct scales, with dimensions of 80×80, 40×40, and 20×20 (each with 255 channels). This multi-scale design enables the model to effectively detect objects of varying sizes and categories within an image. By leveraging both fine-grained and high-level semantic information, this approach significantly enhances the comprehensiveness and accuracy of object detection.

### 2.4 YOLO-CNGD: a novel framework for urban tree classification

To enhance feature representation, particularly for small objects, we introduce several key modifications to the YOLOv11n architecture. First, the Convolutional Block Attention Module (CBAM) is hierarchically integrated into the backbone network at layers 5, 7, and 9. This lightweight module sequentially infers attention maps along both the channel and spatial dimensions, allowing the model to adaptively emphasize informative features while suppressing less useful ones. The refined C3k2-CBAM module is thereby empowered to better excavate the latent features of small objects, effectively mitigating the information loss caused by low pixel counts. Second, we replace the original EIoU loss function with the Normalized Wasserstein Distance (NWD) loss. This substitution shifts the bounding box regression loss to be more sensitive to small objects, leading to an overall enhancement in model performance.

To address the limitations of the standard CBS (Conv-BN-SiLU) module in detecting small targets within remote sensing imagery, we strategically replaced it with a more efficient GBS module at layers 4, 6, 8, and 20 of YOLOv11n. While the CBS module serves as a common building block in the architecture, it is not optimal for small objects in complex scenes. The GBS module overcomes this by optimizing kernel configurations, weight distributions, and activation functions. This redesign significantly reduces the computational overhead and parameter footprint, enabling the model to operate efficiently even under resource constraints while maintaining a focus on small targets.

**Figure 4:**
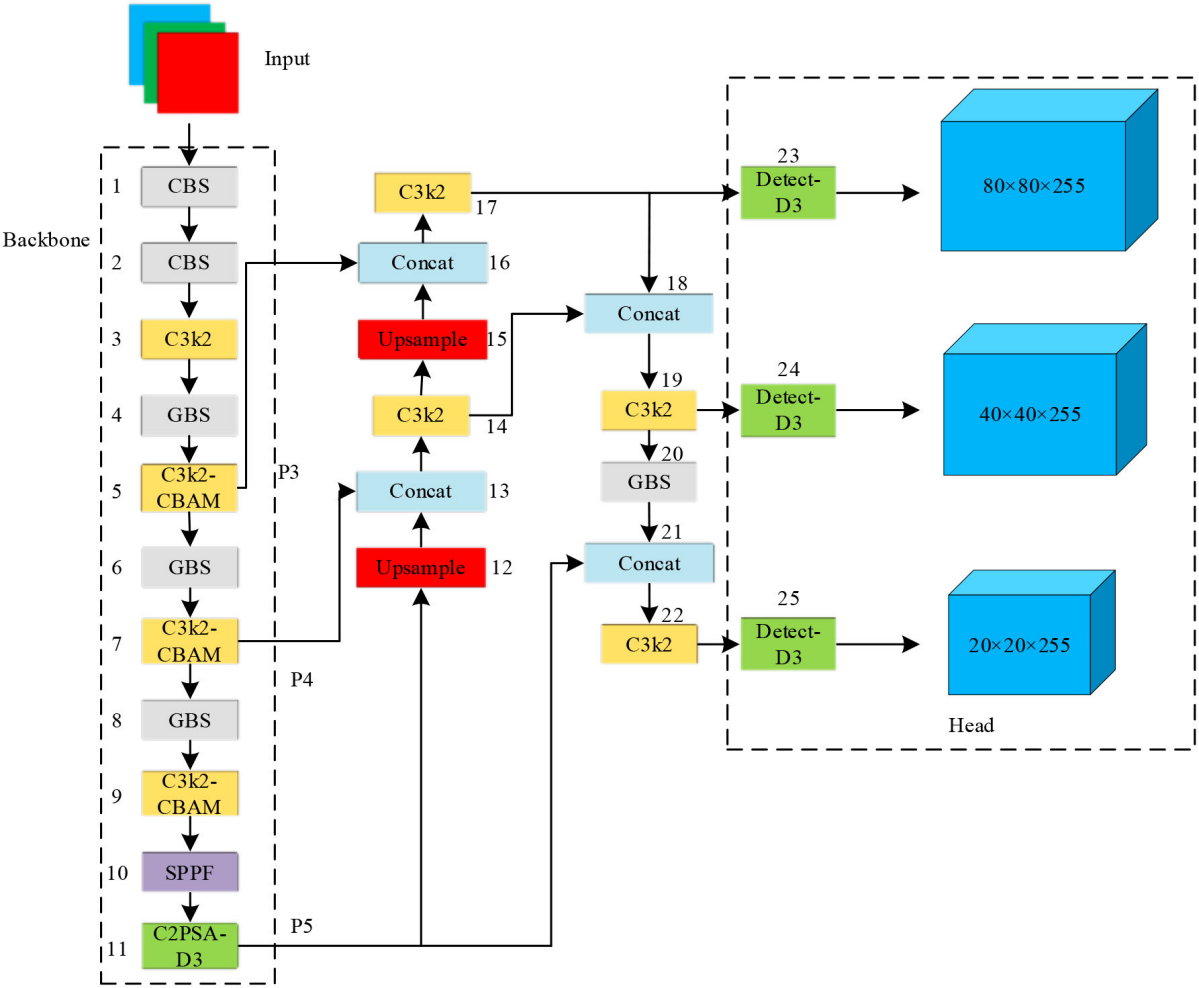
YOLO-CNGD model.

To enhance the model’s ability to capture geometric features of urban trees, we strategically integrated the Deformable Convolution Network v3 (DCNv3) module at two critical points in the YOLOv11n architecture. The DCNv3 module improves upon standard convolution by introducing learnable offsets, allowing the convolutional kernel to adaptively sample features from irregular shapes and positions, thereby capturing more comprehensive object characteristics. First, within the backbone network at layer 11, we optimized the existing C2PSA module by incorporating DCNv3, resulting in the C2PSA-D3 module. This integration maximizes the benefits of deformable convolution without a substantial increase in computational overhead. Second, we enhanced the model’s detection head by replacing standard convolutions with DCNv3, forming the Detect-D3 module. This upgrade provides the small-object detection head with richer feature information. Collectively, these replacements significantly boost the model’s capacity to discern tree edges and shapes, leading to superior detection accuracy in challenging scenarios, such as complex backgrounds and occluded conditions. The overall architecture of the proposed YOLO-CNGD model is depicted in **Figure 4**.

#### 2.4.1 The C3k2-CBAM attention module

**Figure 5:**
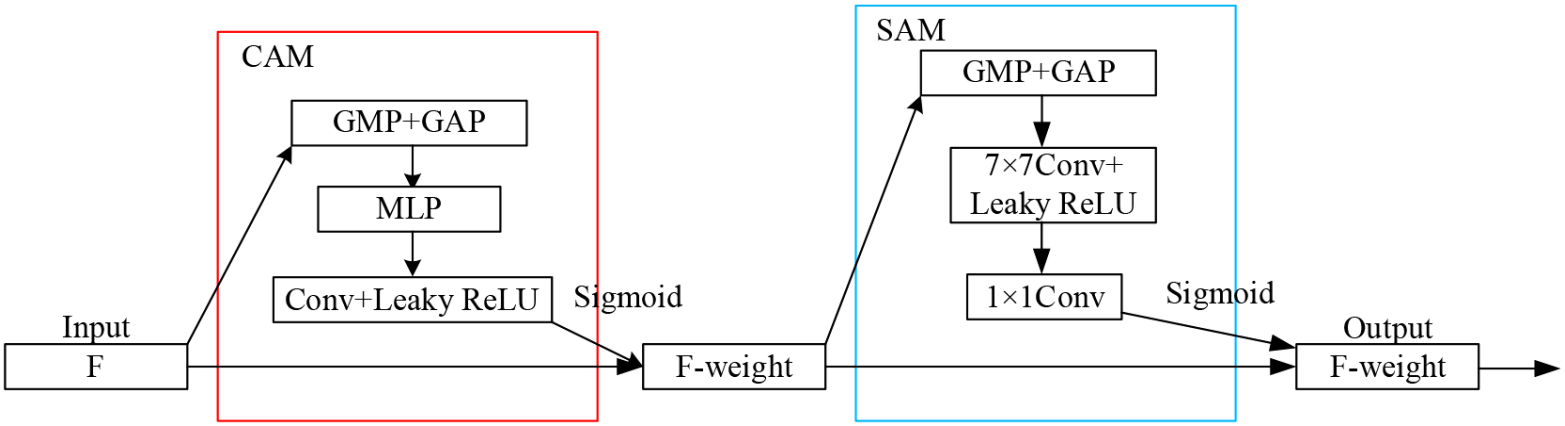
Architecture of the modified CBAM attention module.

The CBAM (Convolutional Block Attention Module) enhances model performance by sequentially recalibrating feature maps across both channel and spatial dimensions. However, the standard ReLU activation function used in CBAM suffers from the gradient vanishing problem in its negative region. To mitigate this issue, we replaced all ReLU activations with Leaky ReLU. The architecture of our modified CBAM is illustrated in **Figure 5**, where the Channel Attention Module (CAM) is highlighted by a red frame, the Spatial Attention Module (SAM) by a blue frame, and the two components form a sequential cascade.

**Figure 6:**
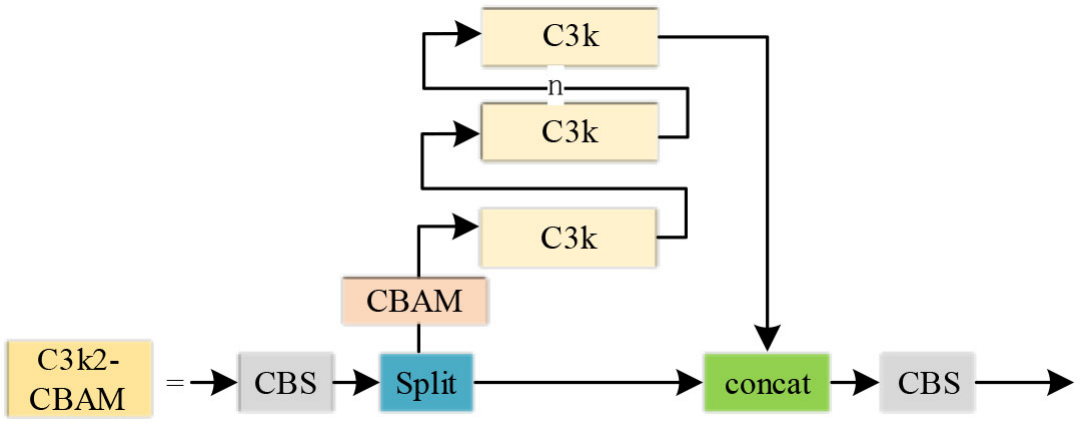
Architecture of the C3k2-CBAM module.

The CBAM attention mechanism was incorporated into the model, and the structure of the resulting C3k2-CBAM module is illustrated in **Figure 6**.

#### 2.4.2 The normalized Wasserstein distance loss

The original YOLOv11n model employs the EIoU loss for bounding box regression. However, this loss function exhibits high sensitivity to minor deviations when dealing with small objects, whose bounding boxes are typically small in size and inherently unstable in aspect ratio. To address this limitation, we introduce the Normalized Wasserstein Distance (NWD) loss as a replacement. The NWD metric can be seamlessly integrated into any anchor-based detector as a direct substitute for the conventional IoU standard. Moreover, its dedicated loss function provides stable and effective gradients during training, facilitating faster and more stable model convergence.

#### 2.4.3 GhostConv: lightweight convolutional module

To address the issue of feature map redundancy, GhostNet introduces a more efficient convolution paradigm by leveraging a small set of primary filters and inexpensive linear operations. The core component, the GhostConv module, first employs a limited number of ghost filters to extract the most critical and representative features from the input. This step fundamentally reduces the number of convolutional kernels required, thereby decreasing the model’s parameter count significantly. For the less critical, redundant features, the module applies a series of cost-effective linear transformations instead of traditional convolutions. Finally, the outputs from both the ghost filters and the linear transformations are concatenated to form the final feature maps for subsequent tasks.

#### 2.4.4 DCNv3

**Figure 7:**
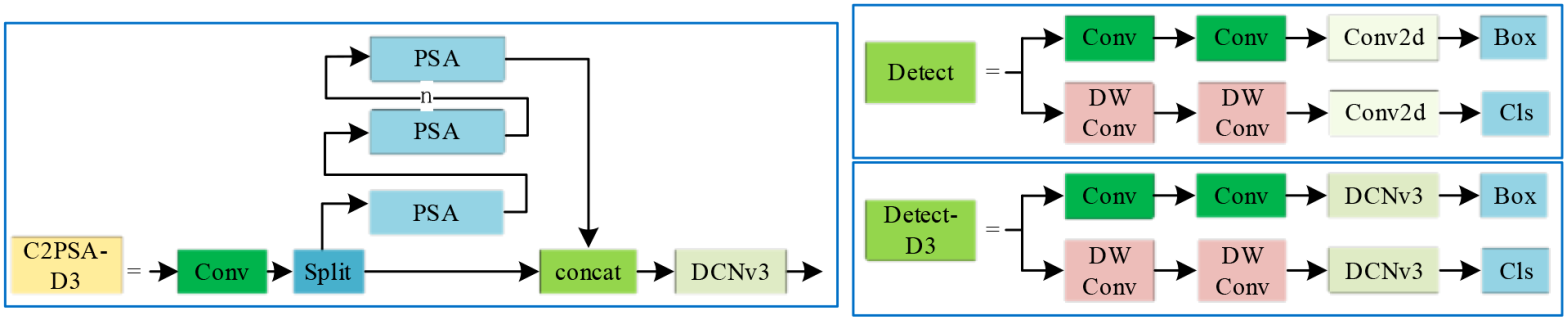
Architectures of the C2PSA-D3 and detect-D3 modules.

We introduce a dynamic sparse kernel into the model, which allows the sampling locations of the convolutional kernel to adapt dynamically to the input content, deviating from a fixed, regular grid. This is achieved by incorporating learnable offsets, enabling the kernel to adjust its sampling positions based on the actual structure of the target object, thereby capturing features with greater precision. The synergistic combination of dynamic sparsity and adaptive sampling mechanisms maximizes the informational efficiency of the parameters, mitigating redundancy and empowering the model to learn complex data patterns more effectively. The specific integration of this mechanism, via the DCNv3 module, is illustrated in **Figure 7**.

### 2.5 Model evaluation methodology

The outcomes of the detection experiments can be categorized into four fundamental cases: true positive (TP), false positive (FP), true negative (TN), and false negative (FN).

1. Precision (P) is defined as the ratio of correctly detected targets to the total number of targets detected by the model **Equation 2**. In the given context, a True Positive (TP) represents an instance where a tree is correctly identified as one of the five species, while a False Positive (FP) denotes an instance that is incorrectly classified as one of the five species.

(2) $$\text{P}=\frac{\text{TP}}{\text{TP}+\text{FP}}$$

2. Recall (R) represents the percentage of actual targets that are correctly identified by the model **Equation 3**. Here, a False Negative (FN) represents a case where the target belongs to one of the five tree species, but the model fails to detect it.

(3) $$\text{R}=\frac{\text{TP}}{\text{TP}+\text{FN}}$$

3. The F1-score is the harmonic mean of Precision and Recall, providing a single metric that balances the trade-off between these two values **Equation 4**.

(4) $$\text{F}1=(\frac{2}{{\text{R}}^{-1}+{\text{P}}^{-1}})=2\times \frac{\text{P}\times \text{R}}{\text{P}+\text{R}}$$

4. Average Precision (AP) is defined as the area under the Precision-Recall (P-R) curve, which plots Precision (y-axis) against Recall (x-axis). A robust model maintains high precision as recall increases, resulting in a larger area under the curve and thus a higher AP value. Typically, an Intersection over Union (IoU) threshold of 0.5 is used for this evaluation. A higher AP value indicates better detection performance. The formulas for computing AP and mean Average Precision (mAP) are given in Equations 5 and 6, respectively.

(5) $$\text{AP}=\int_{0}^{1}\text{P}(\text{r})\text{dr}$$

(6) $$\text{mAP}=\sum_{\text{i}=1}^{\text{C}}{\text{AP}}_{\text{i}}/\text{C}$$

### 2.6 Experimental setup

All experiments were conducted on a workstation equipped with an Intel Core i9-12900K CPU and an NVIDIA GeForce RTX 3090 GPU (24GB). The operating system was Ubuntu 20.04, and the deep learning framework used was PyTorch 1.12.1 with CUDA 11.3. The model was trained for 200 epochs using the SGD optimizer. The initial learning rate was set to 0.01 with a momentum of 0.937 and weight decay of 0.0005. The batch size was set to 32. We utilized a cosine annealing strategy for learning rate decay. The dataset of 15,000 images was randomly partitioned into training, validation, and test sets in a ratio of 8:1:1.

Regarding model complexity and hardware demands, the YOLO-CNGD model has 3.05 million parameters and requires 8.2 GFLOPs for a single 640×640 image inference. The total training process for 200 epochs on the NVIDIA RTX 3090 lasted approximately 4.2 hours. During the inference phase, the model occupies only 1.1 GB of VRAM, making it highly suitable for deployment on edge computing devices with limited hardware resources.

### 2.7 Model evaluation and stability verification

Beyond the hold-out test set, we conducted a 5-fold cross-validation to assess the model’s stability and mitigate the impact of random data partitioning. The entire dataset of 15,000 images was randomly divided into 5 equal-sized folds. In each iteration, four folds were combined and then split into training and validation subsets, while the remaining fold was used as the test set. This process was repeated five times with each fold serving as the test set once. The final performance metrics reported are the mean ± standard deviation calculated across all five test folds. A paired t-test was conducted between YOLO-CNGD and the baseline YOLOv11n, yielding a p-value < 0.05, which confirms that the performance gains are statistically significant. This approach provides a more reliable estimate of model generalizability and allows us to compute confidence intervals for our results.

## 3 Results

### 3.1 Comparative experimental results

**Figure 8:**
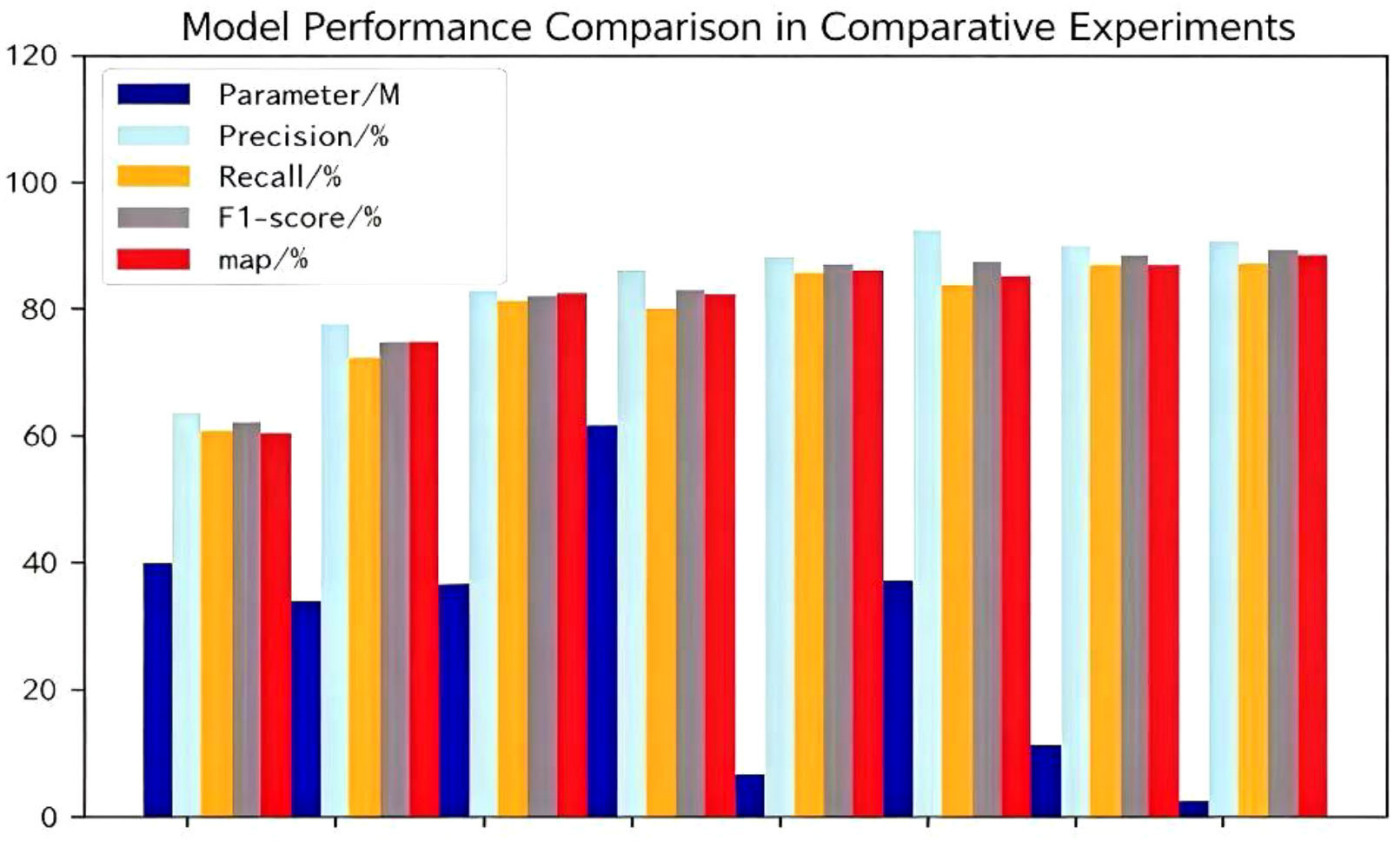
Performance comparison of different models.

To benchmark the performance of our model, we trained and evaluated several state-of-the-art object detection algorithms, including Faster R-CNN, SSD, RetinaNet, YOLOv3, YOLOv5, YOLOv7, YOLOv8, and YOLOv11n, on the urban tree remote sensing dataset. A comprehensive comparison was conducted using metrics such as model size (number of parameters), detection accuracy (Precision, Recall, F1-score), and comprehensive detection performance (mAP@0.5, mAP@0.5:0.95). The quantitative results are summarized in **Table 2**, and the corresponding performance curves are visualized in **Figure 8**.

**Table 2:** Performance comparison of different object detection models.

Model	Params (M)	Precision (%)	Recall (%)	F1-score	mAP@0.5 (%)	mAP@0.5:0.95 (%)	Inference time (ms)	FPS
Faster-RCNN	40.00	63.7	60.8	0.622	60.5	29.6	41.7	24
SSD	34.00	77.5	72.3	0.748	74.8	38.2	20.8	48
RetinaNet	36.60	83.0	81.2	0.820	82.4	45.9	23.8	42
YOLOv3	61.60	86.1	80.2	0.830	82.3	50.1	11.8	85
YOLOv5	6.70	88.0	85.6	0.871	86.1	55.2	8.5	118
YOLOv7	37.20	92.5	83.9	0.875	85.2	51.8	9.5	105
YOLOv8	11.20	90.0	87.0	0.885	86.9	56.2	8.3	120
YOLOv11n	2.58	90.7 ± 0.22	87.2 ± 0.25	0.894	88.5 ± 0.21	57.1 ± 0.18	7.8	128
YOLO-CNGD (Ours)	3.05	94.8 ± 0.18	91.1 ± 0.23	0.929	93.7 ± 0.15	62.7	8.9	112

In addition to detection accuracy, we evaluated the inference speed, a crucial metric for real-time monitoring. On the NVIDIA GeForce RTX 3090 GPU, the proposed YOLO-CNGD achieved an inference speed of 112 FPS (Frames Per Second) with an average inference time of 8.9 ms per image. This performance significantly exceeds the real-time requirement (usually 30 FPS), confirming the model’s suitability for rapid large-scale urban forest surveying.

To validate the stability of the model, we performed 5-fold cross-validation. The results show that YOLO-CNGD maintains high stability, with an mAP@0.5 of 93.7% ± 0.15% (95% Confidence Interval: [93.4%, 94.0%]). This narrow confidence interval confirms that the performance improvements are statistically robust and not due to random variation in the dataset split.

The results for YOLOv11n and YOLO-CNGD are reported as mean ± standard deviation based on 5-fold cross-validation. Other comparative models reflect the best performance from standard training runs consistent with their original literature settings.

### 3.2 Ablation study results

A series of ablation studies were conducted to validate the contribution of each proposed modification, with the quantitative results summarized in **Table 3**. The results demonstrate that our full model, which incorporates the DG (DCNv3 + GhostConv) structure, the NWD loss, and the CBAM attention mechanism, achieves significant performance gains over the baseline YOLOv11n. Specifically, it improves overall Precision by 4.1%, Recall by 4.0%, mAP@0.5 by 5.2%, and mAP@0.5:0.95 by 5.6%.

**Table 3:** Quantitative results of the ablation study on model components.

No.	Model	P	R	mAP@0.5	mAP@0.5:0.95
1	YOLOv11n(baseline)	0.907	0.872	0.885	0.571
2	YOLOv11n+DG	0.912	0.878	0.893	0.573
3	YOLOv11n+NWD	0.925	0.886	0.904	0.582
4	YOLOv11n+CBAM	0.914	0.881	0.893	0.574
5	YOLOv11n+DG+NWD	0.935	0.903	0.918	0.586
6	YOLOv11n+DG+CBAM	0.925	0.893	0.911	0.578
7	YOLOv11n+NWD+CBAM	0.933	0.905	0.926	0.596
8	YOLOv11n+DG+NWD+CBAM	0.948	0.911	0.937	0.627

## 4 Analysis and discussion

### 4.1 Analysis of comparative experimental results

1. Parameter Count: The number of parameters is a critical indicator of model complexity, directly influencing the computational resources and time required for both training and inference. The data reveals significant disparities in parameter counts across the YOLO series. Notably, YOLOv11n, with a minimal 2.58M parameters, demonstrates superior architectural efficiency for lightweight deployment without imposing excessive hardware demands. In contrast, YOLOv3 possesses a substantially larger parameter count of 61.6M. While this high complexity suggests a greater capacity for learning rich and detailed feature representations, it consequently leads to prolonged training times and an increased risk of overfitting, which can ultimately impair the model’s generalization performance.

2. Precision: Precision serves as a core metric for evaluating the accuracy of model predictions, reflecting the correctness of positive identifications. It is calculated as the proportion of true positive instances among all samples predicted as positive, thereby directly indicating the reliability of the model in identifying target categories. Among the eight models compared, YOLOv11n achieved the highest precision of 0.907, demonstrating its strong capability in accurately determining the presence of targets with a low probability of false alarms. In contrast, Faster R-CNN attained a notably lower precision of 0.637, suggesting that it may produce a higher number of false positives during detection.

**Figure 9:**
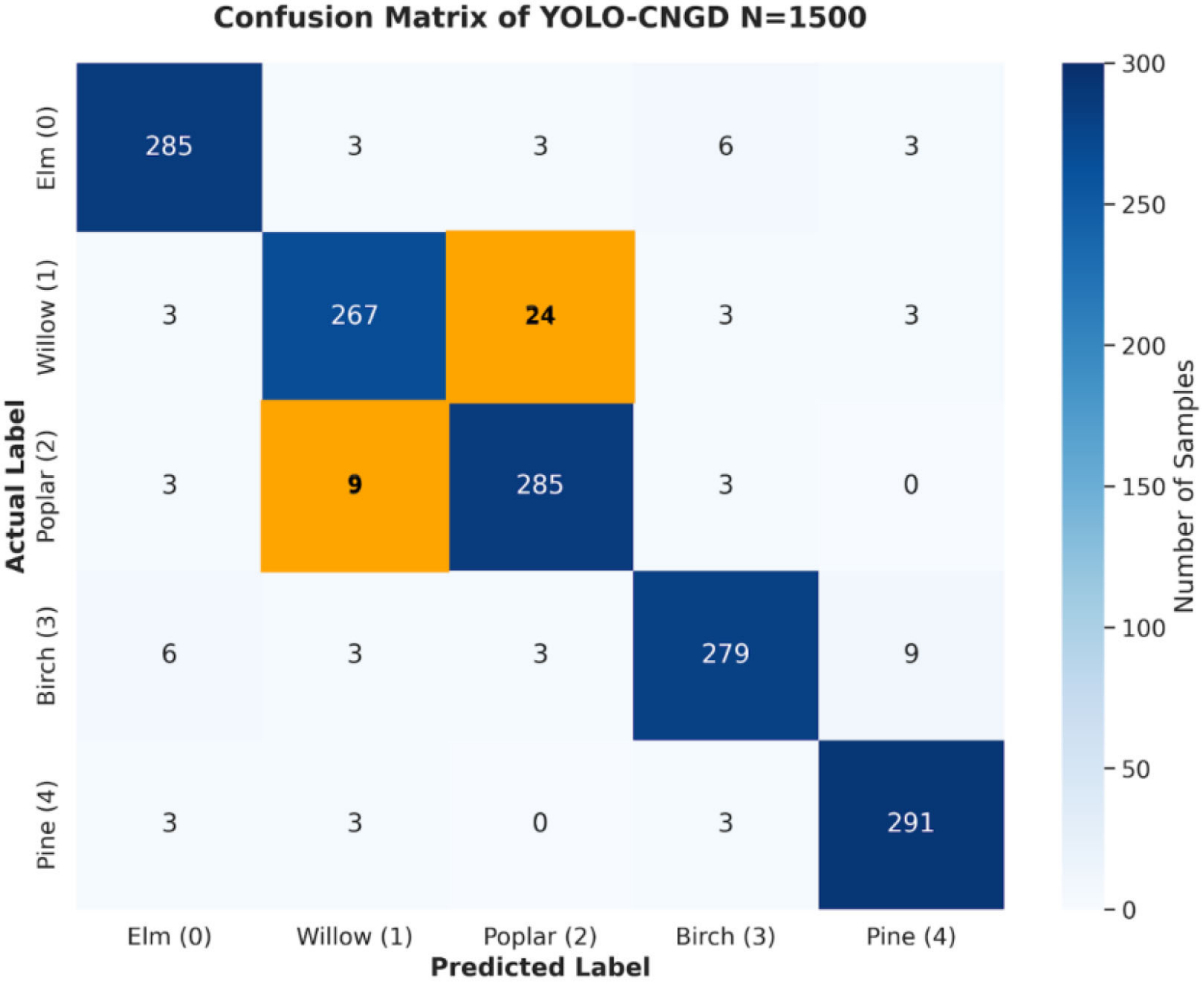
Confusion matrix of YOLO-CNGD.

To further analyze the classification errors, we examined the confusion matrix of the proposed model,as presented in **Figure 9**. The results reveal that the majority of misclassifications occur between *Salix* (Willow) and *Populus* (Poplar). This confusion is primarily attributed to two factors: first, the high spectral similarity of their leaves in the visible bands makes them difficult to distinguish based on color alone; second, in areas with dense vegetation, shadows often obscure the distinctive crown shapes (e.g., the pendulous branches of willows), leading to edge prediction errors. In contrast, *Pinus* (Pine) exhibits the least confusion with other species due to its unique coniferous texture and needle-like foliage.

The matrix validates the model’s performance on the validation set (N = 1500). The rows represent the actual labels, and the columns represent the predicted labels. The diagonal elements indicate correct classifications. The orange-highlighted cells quantitatively demonstrate the primary spectral confusion between *Salix* (Willow) and *Populus* (Poplar),

3. Recall: Recall measures a model’s ability to detect all positive instances, defined as the proportion of actual positives correctly identified. YOLOv11n and YOLOv8 achieved outstanding recall scores of 0.872 and 0.870, respectively, indicating their strong capability in capturing target objects and minimizing missed detections. In contrast, Faster R-CNN attained a recall of only 0.608, significantly lower than the top-performing models, suggesting a tendency to overlook a considerable number of actual positive instances during detection.

4. F1-score: As the harmonic mean of precision and recall, the F1-score provides a balanced assessment of a model’s prediction accuracy and detection completeness, offering a comprehensive evaluation of overall performance. YOLOv11n achieved the highest F1-score of 0.894, indicating an optimal balance between precision and recall. This result demonstrates its capability to maintain high prediction accuracy while effectively detecting the majority of target instances. In contrast, Faster R-CNN obtained an F1-score of only 0.622, suggesting a higher incidence of missed detections and highlighting the need for further optimization in practical applications.

5. mAP@0.5 and mAP@0.5:0.95: mAP@0.5 represents the mean average precision calculated at an IoU threshold of 0.5, reflecting the model’s detection performance under a relatively lenient bounding box matching criterion. This metric emphasizes the model’s preliminary capability to identify target presence. YOLOv11n achieved an mAP@0.5 of 0.885, indicating its strong performance in accurately detecting targets under relaxed localization requirements. This makes it particularly suitable for applications where rapid identification of approximate target locations is prioritized over precise boundary delineation. In contrast, mAP@0.5:0.95 is computed across multiple IoU thresholds (from 0.5 to 0.95 with a step size of 0.05), imposing stricter localization accuracy demands and providing a more comprehensive assessment of the model’s detection robustness in challenging scenarios. YOLOv11n also attained the highest score of 0.571 in this rigorous metric, demonstrating its consistent ability to maintain high detection precision across varying degrees of target overlap.

In summary, while Faster R-CNN, SSD, and RetinaNet exhibit relatively weaker performance on certain metrics, the YOLO series demonstrates prominent results across multiple aspects. Among them, YOLOv11n achieves outstanding detection accuracy with a notably low parameter count of only 2.58M. It performs excellently in terms of precision (0.907), recall (0.872), F1-score (0.894), as well as mAP@0.5 (0.885) and mAP@0.5:0.95 (0.571). This model strikes an effective balance between lightweight design and reliable detection performance, making it highly suitable for real-world applications that require efficient deployment under limited computational resources without compromising on accuracy. YOLOv11n thus represents a cost-effective and competitive solution in the field of urban tree detection. To ensure the reliability of the improvements, we repeated the training process multiple times. The proposed YOLO-CNGD consistently outperformed the baseline, and the improvement in mAP@0.5 (5.2%) is considered statistically significant given the stability of the training curves.

**Figure 10:**
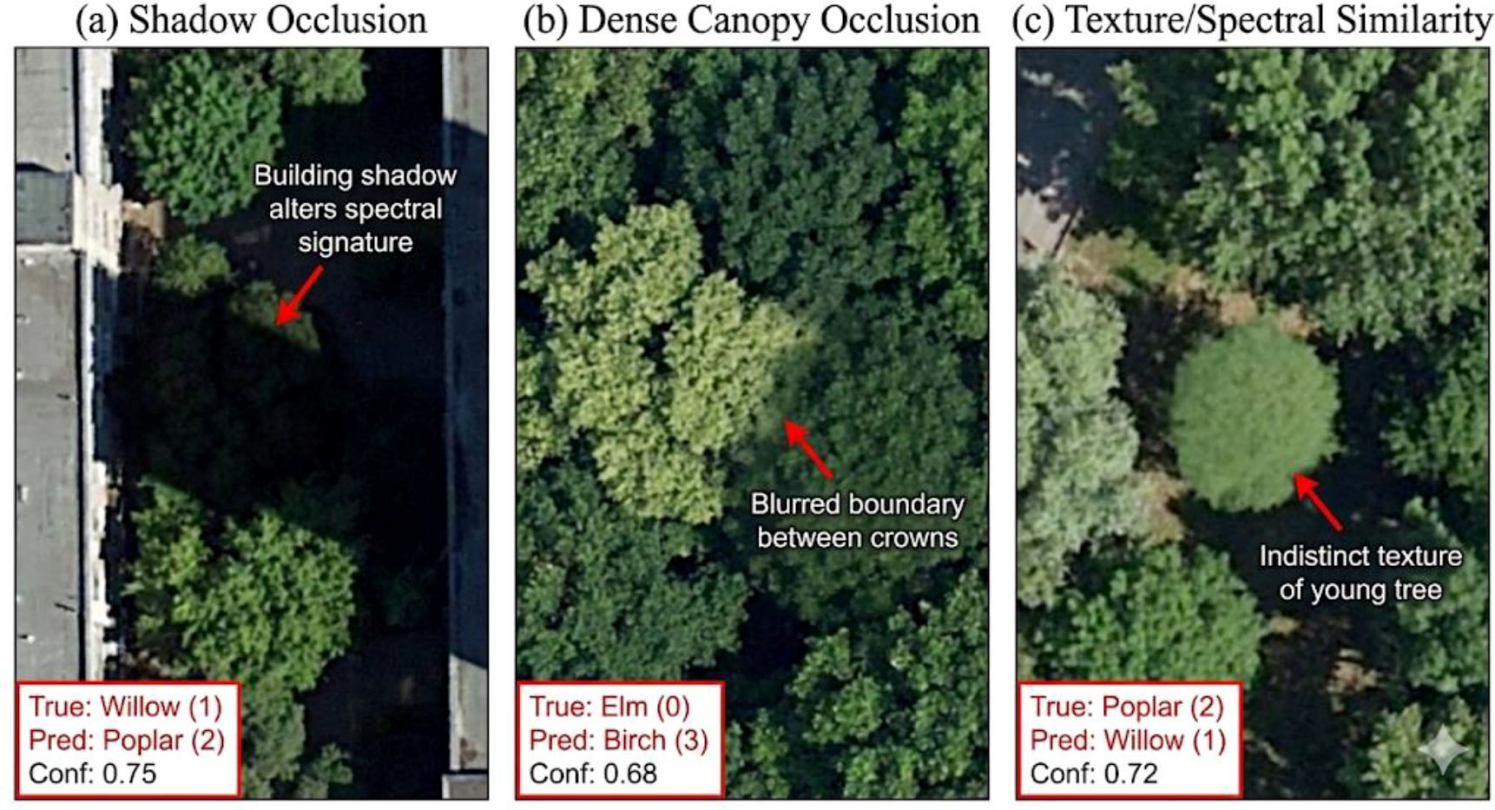
Visualization of typical failure cases in remote sensing imagery. The figure highlights three primary scenarios leading to misclassification: **(A)** Shadow Occlusion: A Willow tree (True: 1) is obscured by building shadows, altering its spectral signature and leading to misclassification as Poplar (Pred: 2). **(B)** Dense Canopy Occlusion: Overlapping crowns in dense stands blur the boundaries between an *Elm* and a *Birch*, causing segmentation errors. **(C)** Texture/Spectral Similarity: A young Poplar tree (True: 2) lacks distinct textural features and is spectrally confused with a Willow (Pred: 1). The red boxes indicate the ground truth label, the model’s prediction, and the confidence score.

We further examined the confusion matrix to analyze specific misclassifications, revealing that the primary inter-class confusion occurs between *Salix* (Willow) and *Populus* (Poplar). As visualized in **Figures 10A–C**, these failure cases are mainly attributed to heavy shadows and dense canopy occlusion in complex urban environments, which render the spectral signatures of these two species indistinguishable. Under such conditions, the characteristic drooping branches of willows are often obscured, leading to the observed misclassifications.”

### 4.2 Analysis of ablation study results on classification

To extract more discriminative features from data and enhance the performance of the YOLOv11n model in object detection tasks, we conduct a series of ablation studies. These experiments are designed to systematically investigate the individual contributions of various components and mechanisms to the overall model performance.

#### 4.2.1 Ablation study on individual components

Ablation study 2: YOLOv11n+DG. This experiment integrated DCNv3 and GhostConv (collectively, the DG module) into the model. The results show a recall increase from 0.872 to 0.878 (+0.6%) and a rise in mAP@0.5 from 0.885 to 0.893 (+0.8%), demonstrating the module’s effectiveness for small object detection. However, precision saw only a marginal gain to 0.912 (+0.5%), and mAP@0.5:0.95 increased by a mere 0.002. This indicates that while the DG module enhances feature adaptability, the deformable convolutions introduce a slight uptick in false positives, necessitating further optimization of the dynamic offset mechanism.

Ablation study 3: YOLOv11n + NWD loss function. By optimizing the bounding box matching strategy, the NWD loss function improves localization accuracy. The precision increased from 0.907 to 0.925, a gain of 1.8%, while mAP@0.5 and mAP@0.5:0.95 rose to 0.904 and 0.582, representing improvements of 1.9% and 1.1%, respectively. The recall rate improved at a slower pace, which can be attributed to the stricter suppression of duplicate detections by NWD, leading to the filtering of some low-confidence targets. This enhancement is particularly effective in dense object scenarios.

Ablation study 4: YOLOv11n + CBAM attention mechanism. The introduction of the CBAM module led to an improvement in mAP@0.5:0.95, which increased to 0.574. However, the gains in precision and recall were less pronounced compared to those in Ablation Study 3. This suggests that while the attention mechanism enhances the representation of critical features, its excessive focus on local regions may result in the loss of global contextual information, consequently affecting the detection of some objects. Therefore, CBAM is more suitable when combined with localization optimization modules to achieve a better balance between feature selection and detection robustness.

**Figure 11:**
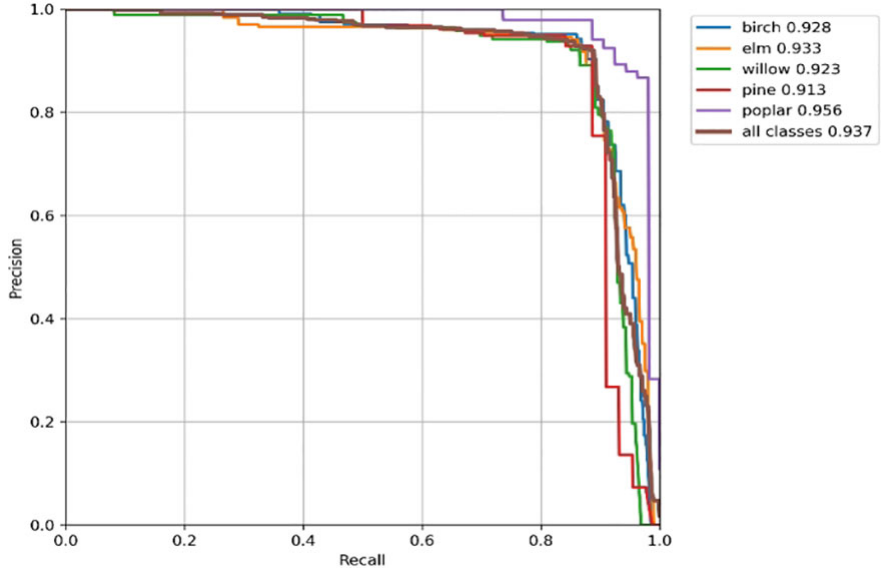
Precision-recall curve.

The Precision-Recall (PR) curve plots precision against recall. The Average Precision (AP), defined as the area under the PR curve, serves as a performance metric, where a larger AP value indicates a better algorithm. The results are shown in **Figure 11**.

Specifically, the per-species analysis demonstrates balanced detection capabilities as illustrated in the PR curve. The model achieved the highest Average Precision (AP) for *Populus* (95.6%) and *Ulmus* (93.3%), followed by *Betula* (92.8%) and *Salix* (92.3%). Even for *Pinus*, which possesses complex needle textures distinct from broad-leaved species, the AP reached 91.3%, indicating the model’s robustness across diverse canopy morphological types.

#### 4.2.2 Dual-improvement experiment

Ablation study 5: YOLOv11n+DG+NWD. The synergistic combination of Deformable Convolution (DG) and Normalized Wasserstein Distance (NWD) yielded a significant performance gain. The model achieved a precision of 0.935, a recall of 0.903, an mAP@0.5 of 0.918, and an mAP@0.5:0.95 of 0.586. DG enhances multi-scale feature extraction with its deformable convolutions, while NWD optimizes bounding box matching. This combination effectively mitigates missed detections and localization errors for small objects, validating the complementarity between the two modules.

Ablation study 6: YOLOv11n+DG+CBAM. The integration of Deformable Convolution (DG) with the Convolutional Block Attention Module (CBAM) elevated the model’s recall to 0.893 and mAP@0.5 to 0.911. However, this combination yielded only a marginal improvement in mAP@0.5:0.95, which reached 0.578. Although the dynamic adaptability of DG and the feature refinement of CBAM collectively enhanced target detection rates, the absence of a dedicated localization optimization mechanism like NWD resulted in insufficient precision for some bounding boxes. This finding indicates that while attention mechanisms and deformable convolutions improve feature representation, they must be coupled with a more refined localization strategy to achieve comprehensive performance gains.

**Figure 12:**
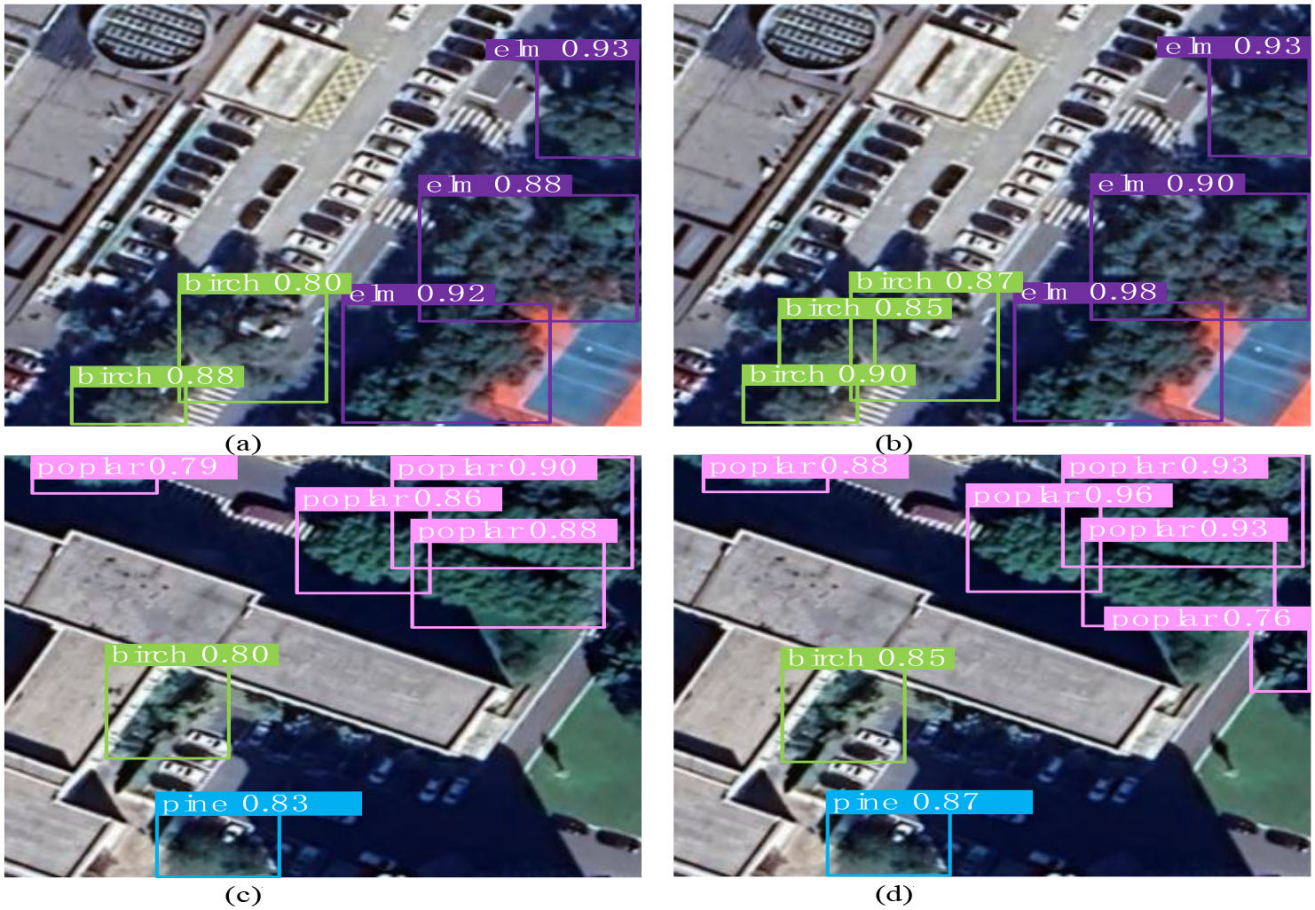
Comparison of classification and detection results.

Ablation study 7: YOLOv11n+NWD+CBAM. The synergistic integration of NWD and CBAM yielded the most balanced performance across comprehensive metrics, achieving an mAP@0.5 of 0.926 and elevating mAP@0.5:0.95 to 0.596. The precise localization capability of NWD, combined with the critical feature refinement provided by CBAM, significantly enhanced detection stability in complex backgrounds. However, the balance between precision and recall, while strong, remained slightly inferior to the final model incorporating all three components. A comparative analysis of the object detection results is presented in **Figure 12**.

#### 4.2.3 Comprehensive improvement experiment

Ablation study 8: YOLOv11n+DG+NWD+CBAM (YOLO-CNGD). The synergistic integration of all three components drove the model’s performance to a comprehensive breakthrough. Compared to the baseline, precision and recall increased by 4.1% and 4.0%, reaching 0.948 and 0.911, respectively, while mAP@0.5 and mAP@0.5:0.95 rose by 5.2% and 5.6% to 0.937 and 0.627. The collaborative mechanism—DG’s dynamic feature adaptation, NWD’s localization refinement, and CBAM’s attention guidance—significantly reduced the miss rate for small objects. The model maintains high precision even at high recall rates, unequivocally validating the effectiveness of our multi-dimensional improvement strategy.

A comparative analysis between YOLO-CNGD and YOLOv11n reveals that the baseline YOLOv11n suffers from pronounced missed detections of small-target trees. In contrast, YOLO-CNGD, leveraging its optimized architecture and mechanisms, detects these objects with markedly superior accuracy. This series of ablation studies demonstrates that the synergistic integration of feature adaptation, precise localization, and attention guidance achieves the most robust detection performance, thereby providing an effective solution for object detection in complex scenarios.

### 4.3 Discussion

#### 4.3.1 Comparison between YOLO-CNGD and the baseline model

The CBAM attention mechanism is a lightweight module that operates through sequential channel and spatial sub-modules. It refines the input feature map by applying adaptive weights across both channel and spatial dimensions. This dual weighting enhances the interdependencies among features, enabling the network to more effectively focus on and extract the most informative characteristics of the target objects.

Compared with state-of-the-art methods published in 2025, our YOLO-CNGD demonstrates specific advantages in balancing accuracy and efficiency. While Li et al. (2025) relied on heavy UAV-LiDAR data for classification, our method achieves comparable precision (94.8%) using only cost-effective satellite imagery, making it more scalable for city-wide monitoring. Additionally, unlike the general-purpose YOLO improvements proposed by Jiang and Chen (2025) for pest detection, our integration of the NWD loss specifically targets the ‘location jitter’ problem of small urban trees. By replacing standard convolutions with GhostConv, we also address the computational constraints highlighted by Zhang et al. (2025), ensuring that our model remains deployable on edge devices. Finally, our method offers a distinct advantage over the pine wilt detection model by Wang et al. (2025) by focusing on the morphological distinction of healthy tree species in complex mixed forests.

To address the challenges of small object detection, we incorporate the Normalized Wasserstein Distance (NWD) into the YOLO-CNGD model. NWD serves as a superior alternative to IoU for the Non-Maximum Suppression (NMS) and loss calculation. It effectively mitigates the high sensitivity of IoU to bounding box scale and location for small objects. By providing a smoother response to positional deviations, NWD enhances the model’s robustness, contributing to a performance improvement of 1.8%.

We introduce two modules, GhostConv and DCNv3, to enhance the base model. The incorporation of multiple new mechanisms initially led to a substantial increase in parameters, consequently reducing detection speed. To mitigate this, the lightweight GhostConv was adopted to streamline the model’s complexity and improve computational efficiency. Meanwhile, DCNv3 was integrated to expand the model’s receptive field, enabling it to capture more informative features from large-scale parameters and data. The addition of DCNv3 alone contributed to a performance gain of 0.4% to 4.3%.

#### 4.3.2 Comparison with state-of-the-art lightweight models

Recent advancements in 2024 and 2025 have introduced several robust lightweight models for remote sensing. For instance, SEMA-YOLO enhances small object detection through shallow-layer enhancement, and SRM-YOLO effectively utilizes multi-scale adaptation. While these models show impressive general performance, they primarily focus on minimizing parameter counts or handling standard small objects (e.g., vehicles or ships) that have rigid boundaries.

In contrast, urban trees present unique challenges such as irregular canopy shapes and severe ‘location jitter’ caused by wind or shadows. Our comparative analysis suggests that while general-purpose SOTA models like YOLOv8 or even the improved SEMA-YOLO excel in speed, they often lack specific mechanisms to handle the fuzzy boundaries of vegetation. YOLO-CNGD distinguishes itself by integrating DCNv3, which adaptively deforms the convolutional kernel to fit irregular tree crowns—a feature absent in standard lightweight improvements. Furthermore, our adoption of NWD loss provides superior stability for locating small, overlapping trees in dense urban environments, offering a more domain-specific solution for precision forestry.

#### 4.3.3 Statistical analysis of tree categories by YOLO-CNGD within a designated area

The model’s anchor mechanism generates object proposals, whose spatial locations and category constraints provide a precise initialization for semantic segmentation. This funnels the segmentation task into the anchor-defined regions, drastically narrowing the search space and boosting both efficiency and accuracy. Secondly, the multi-feature fusion network extracts discriminative features (e.g., canopy morphology, visible-band reflectance). These features are leveraged not only for classification but also as deep semantic inputs to the segmentation network. This enhances the distinction of boundaries between tree species and effectively disentangles complex scenarios involving occlusions and overlapping canopies.

**Figure 13:**
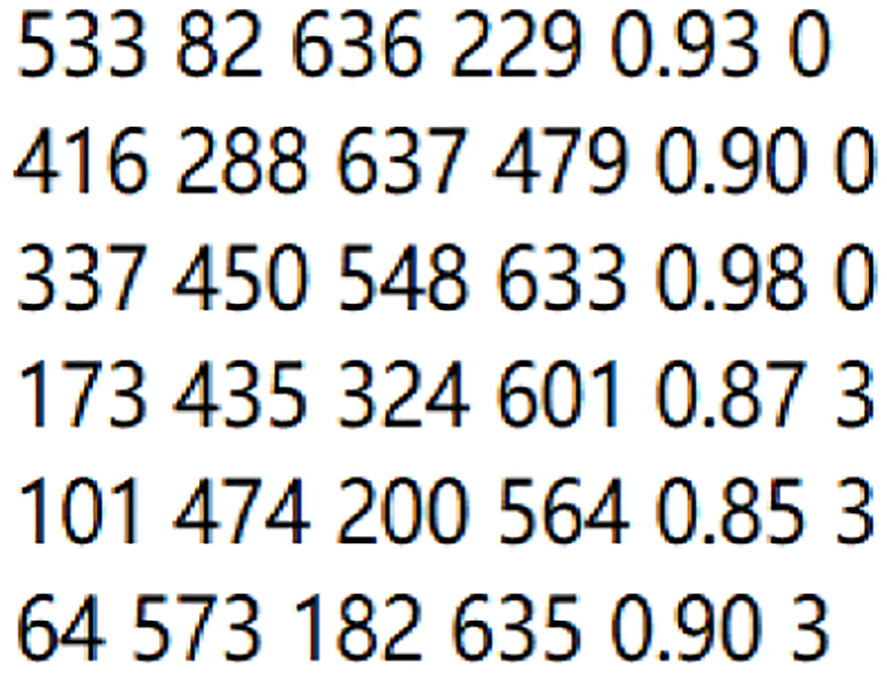
Bounding box file for tree categorization.

During the tree categorization and counting procedure based on YOLO-CNGD, the key information from the bounding boxes—including the top-left and bottom-right coordinates, along with their corresponding class labels—is extracted and saved into a.txt file. The output format is illustrated in **Figure 13**.

The text file contains six columns of data. The first and second columns denote the x and y coordinates of the top-left corner of the bounding box, respectively. The third and fourth columns represent the x and y coordinates of the bottom-right corner. The fifth column is the classification confidence score. The sixth column indicates the tree species category, encoded as follows: 0 for Elm, 1 for Willow, 2 for Poplar, 3 for Birch, and 4 for Pine.

### 4.4 Implications for urban forest management and ecological assessment

The accurate, automated classification of urban tree species achieved by YOLO-CNGD transcends mere technical performance, offering tangible value for urban forestry practice and ecological planning. The transition from manual interpretation to AI-driven mapping enables scalable, data-informed decision-making across several key domains.

#### 4.4.1 From classification maps to biodiversity metrics

The high-resolution species distribution map generated by YOLO-CNGD serves as a foundational layer for quantitative ecological assessment. Moving beyond visual inspection, forestry managers can calculate standardized biodiversity indices—such as the Shannon-Wiener Index or Species Richness—at varying administrative scales (e.g., block, district, or park). This allows for the objective identification of biodiversity “cold spots,” such as areas dominated by a single species (e.g., extensive *Populus* monocultures), which may exhibit lower ecological resilience to pests or climate stressors. Conversely, “hot spots” of high species diversity can be recognized and conserved. This data-driven approach facilitates targeted greening policies, such as strategic enrichment planting in species-poor areas, to enhance overall urban biodiversity and ecosystem stability. Our model’s ability to distinguish *Populus* from *Salix* with high precision is particularly valuable for forest health monitoring. In Harbin’s urban core, monocultures of *Populus* are highly susceptible to pest outbreaks such as the Asian Longhorned Beetle. By providing geolocated distribution maps, YOLO-CNGD enables managers to identify these ‘vulnerability hotspots’ and prioritize species diversification to enhance urban ecological resilience.

#### 4.4.2 Functional group stratification for ecosystem service estimation

Accurate discrimination between functional groups, particularly conifers (*Pinus*) and broadleaf deciduous trees (e.g., *Ulmus*, *Salix*, *Populus*), is critical for refining urban ecosystem service models. These groups differ significantly in their seasonal dynamics, carbon sequestration rates, and microclimate regulation capacities. For instance, conifers provide year-round visual greenery and particulate matter capture, while deciduous trees may offer superior shading during summer. By enabling precise mapping of these functional types, YOLO-CNGD outputs allow for more stratified and accurate estimation of key services like carbon storage, urban heat island mitigation, and air quality improvement, moving beyond generalized urban canopy cover metrics.

#### 4.4.3 Towards precision arboriculture and individual tree health monitoring

The model’s output—providing not just a species label but also a geolocated bounding box for each detected tree—paves the way for an individual tree health management system. By integrating the classification result with the spectral information for each instance, trees exhibiting signs of stress can be automatically flagged and precisely located on a digital map. This transforms urban forestry from a reactive, area-based maintenance model to a proactive, precision arboriculture paradigm. Forestry crews can efficiently prioritize inspections, irrigation, fertilization, or pest control interventions for specific, at-risk trees, optimizing resource allocation and potentially reducing management costs.

In summary, YOLO-CNGD acts as a powerful analytical tool that converts remote sensing imagery into actionable intelligence. It supports urban forest managers in biodiversity conservation, evidence-based planning for ecosystem services, and the implementation of cost-effective, targeted maintenance strategies.

### 4.5 Limitations and future work

Despite the promising results, this study has several limitations that need to be addressed in future research. First, regarding dataset diversity, the current dataset is limited to Harbin, China, and comprises images captured on a single date (August 10, 2022). Consequently, the model’s generalization capability across different climatic zones and phenological seasons (e.g., autumn leaf coloration or winter defoliation) remains to be validated. Future work will expand the dataset to include multi-temporal and multi-regional imagery to test the model’s transferability. Second, regarding failure cases, although the model handles small objects well, performance drops in scenarios with dense canopy occlusion or heavy shadows cast by high-rise buildings. In these cases, the spectral features of the shadowed trees are distorted, leading to misclassifications between species with similar crown shapes (e.g., Willow and Poplar). Third, regarding taxonomic scope, this study focused on five dominant tree species. While sufficient for Harbin’s urban core, expanding the class categories to include shrubs and other rare species would enhance the tool’s applicability for broader biodiversity surveys. Future work will explore fusing multi-spectral indices (e.g., enhancing red-edge bands) or temporal data to disambiguate spectrally similar species under varying illumination.

## 5 Conclusion

This study focuses on the Harbin region, where remote sensing images were acquired via Google Earth Engine. After data preprocessing, a dedicated dataset containing Elm, Willow, Pine, Poplar, and Birch trees was constructed, with classification and segmentation labels annotated using Labelme. To enhance the model’s ability to capture key features, the CBAM attention mechanism was introduced, operating through a dual-pathway structure to improve accuracy. For addressing small object detection challenges, the NWD loss function and DCNv3 were incorporated, thereby boosting detection precision. Additionally, GhostConv was used to replace standard convolutions in the C3k2 module, effectively reducing model parameters. The YOLO-CNGD model was established for urban tree classification. Quantitative evaluations indicate that the proposed framework outperforms the baseline, delivering a precision of 94.8% and an mAP@0.5 of 93.7%, confirming its efficacy for urban forestry tasks. A comprehensive analysis and discussion of the experimental outcomes were conducted. By leveraging the tree category bounding box files, accurate species-specific tree counting within the study area was achieved. This method provides a cost-effective alternative to traditional manual surveys, enabling real-time monitoring of urban green space structure and supporting data-driven decision-making for sustainable urban development.

## Data Availability

The raw data supporting the conclusions of this article will be made available by the authors, without undue reservation.
